# Unmasking cerebellar disease: functional neurologic disorder as a precursor to spinocerebellar ataxia type 8

**DOI:** 10.1007/s10072-025-08553-5

**Published:** 2025-10-02

**Authors:** Sopiko Jimsheleishvili, Domini Crandon, Jason Margolesky

**Affiliations:** Department of Neurology, University of Miami Miller School of Medicine, 1150 NW 14thStreet, Miami, Fl 33136 USA

**Keywords:** Functional neurological disorder, Spinocerebellar ataxia, SCA8

At age 54, a woman with no personal or family history of neurologic disease developed a gait disturbance marked by exaggerated extension of the left quadriceps—appearing as if she were “kicking” with each step. Her symptoms improved with walking backward, barefoot, or when distracted by performing serial 7 s during ambulation. Brain MRI performed at symptom onset revealed moderate, predominantly midline, cerebellar atrophy (Fig. 1). At the time, she had no clinical signs of ataxia, and her SARA score was 0. The distractibility and incongruence of her gait supported a diagnosis of functional neurological disorder (FND). On the Simplified Functional Movement Disorders Rating Scale (S-FMDRS), she scored 3, reflecting a mild gait disturbance present during standard assessment but normalizing with distraction. Her walking improved significantly with intensive physiotherapy focused on FND.

**Fig. 1:**
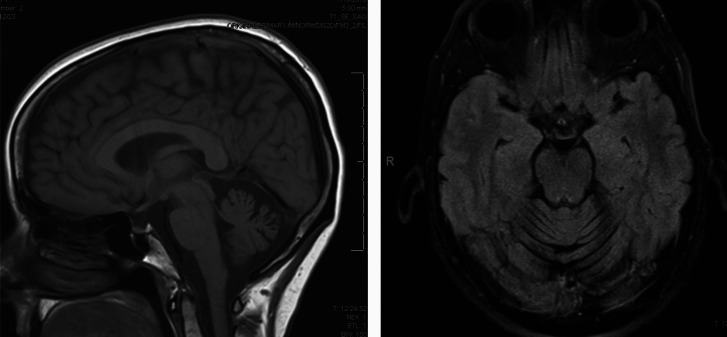
Sagittal T1 MRI and axial FLAIR MRI brain sequences show cerebellar atrophy in a patient with functional gait disorder without overt cerebellar symptoms

At age 59, she developed cerebellar dysarthria—her first overt motor manifestation of cerebellar degeneration—as well as anxiety, depression, and mood lability. These developments prompted reevaluation of her cerebellar atrophy. Autoimmune and paraneoplastic antibody panel testing was unrevealing. A genetic ataxia panel showed 112 and 29 CTG repeats in the ATXN8OS gene, confirming a diagnosis of spinocerebellar ataxia type 8 (SCA8). A heterozygous missense VUS in CACNA1A (c.3045G > C, p.Glu1015Asp) was also detected, though its significance remained nebulous.

At age 61, she had ongoing dysarthria, mild bilateral intention tremors, impaired tandem gait, and normal ocular movements. Notably, her functional gait pattern re-emerged during follow-up as it was exacerbated by the stress of having the examination videotaped (Video 1).

This case highlights a rare presentation of FND which preceded overt cerebellar symptoms in a patient with SCA8. While cerebellar atrophy was present at initial evaluation, classic signs of cerebellar disease did not appear for five years. This supports the hypothesis that FND may represent a prodromal phenotype of an underlying neurodegenerative disease. These findings also raise the possibility that cerebellar degeneration can predispose to FND. FND has also been reported in an individual with SCA6, but in that case the FND was not prodromal to the ataxia [1].

SCA8 is an autosomal dominant condition. With maternal inheritance, genetic anticipation with CTG repeat expansion is more likely. Interestingly, increased interruptions in the CTG repeat expansion, more so than the repeat length itself, predicts earlier age of onset. [2] Phenotypically, SCA8 can present with ataxia, upper motor neuron signs including spasticity, cognitive symptoms, mood disorders, personality changes, and even paroxysmal kinesigenic dyskinesia (PKD; brief, action-induced choreiform or dystonic movements) have been described [3].

Our report mirrors observations in Parkinson’s disease, where FND has been reported to occur before or after the onset of classic Parkinson’s motor signs [4, 5]. Prior studies have found that some patients with “psychogenic” parkinsonism have abnormal Dopamine Transport (DAT) scans, suggesting degeneration of dopaminergic cells, and providing evidence of concurrent degenerative parkinsonism [4]. FND could be considered a potential prodromal feature of Parkinson’s disease [6].

In our patient, FND preceded cerebellar dysarthria and appendicular ataxia, but the FND did not precede cerebellar atrophy. Functional gait disorders can be recognized by inconsistency in gait symptoms and incongruence with understood anatomy and pathophysiology [7]. Early cerebellar dysfunction associated with cerebellar atrophy may contribute to FND development in a patient with a conducive biopsychosocial milieu of FND risk factors.

Functional neurologic symptoms may reflect early dysfunction in cerebellar-affective circuits as a corollary to the cerebellar cognitive affective syndrome (CCAS) [8]. The CCAS can involve impaired executive function, personality changes, altered mood and emotional regulation [9]. SCA8 can manifest cognitive and emotional symptoms [10] and the cerebellum’s role in emotional regulation, automatic motor control, and network connectivity with limbic and striatal systems further supports this potential FND-CCAS relationship.

Functional Neurologic Disorder can be prodromal to ataxia in SCA8. Recognizing that FND could be a harbinger of neurodegeneration, patients with FND should be followed over time to monitor for the development of non-functional symptoms including parkinsonism and cerebellar signs. This may be especially true in the setting of imaging biomarkers, such as cerebellar atrophy (or an abnormal DAT scan). Detecting these potential comorbid conditions will allow for early appropriate management and hopefully reduced morbidity.

## Supplementary Information

Below is the link to the electronic supplementary material.

Supplementary file1 (MOV 91717 KB)
